# MiR-182-5p Is Upregulated in Hepatic Tissues from a Diet-Induced NAFLD/NASH/HCC C57BL/6J Mouse Model and Modulates Cyld and Foxo1 Expression

**DOI:** 10.3390/ijms24119239

**Published:** 2023-05-25

**Authors:** Chiara Compagnoni, Roberta Capelli, Veronica Zelli, Alessandra Corrente, Davide Vecchiotti, Irene Flati, Mauro Di Vito Nolfi, Adriano Angelucci, Edoardo Alesse, Francesca Zazzeroni, Alessandra Tessitore

**Affiliations:** 1 Department of Biotechnological and Applied Clinical Sciences, University of L’Aquila, Via Vetoio, 67100 L’Aquila, Italy; chiara.compagnoni@univaq.it (C.C.); roberta.capelli@graduate.univaq.it (R.C.); alessandra.corrente@graduate.univaq.it (A.C.); davide.vecchiotti@univaq.it (D.V.); irene.flati@graduate.univaq.it (I.F.); mauro.divitonolfi@univaq.it (M.D.V.N.); adriano.angelucci@univaq.it (A.A.); edoardo.alesse@univaq.it (E.A.); francesca.zazzeroni@univaq.it (F.Z.);; 2 Center for Molecular Diagnostics and Advanced Therapies, University of L’Aquila, Via Petrini, 67100 L’Aquila, Italy

**Keywords:** miR-182-5p, Cyld, Foxo1, NAFLD, NASH, HCC, biomarkers, therapeutic target

## Abstract

Non-alcoholic fatty liver disease (NAFLD) is considered a relevant liver chronic disease. Variable percentages of NAFLD cases progress from steatosis to steatohepatitis (NASH), cirrhosis and, eventually, hepatocellular carcinoma (HCC). In this study, we aimed to deepen our understanding of expression levels and functional relationships between miR-182-5p and Cyld-Foxo1 in hepatic tissues from C57BL/6J mouse models of diet-induced NAFL/NASH/HCC progression. A miR-182-5p increase was detected early in livers as NAFLD damage progressed, and in tumors compared to peritumor normal tissues. An in vitro assay on HepG2 cells confirmed *Cyld* and *Foxo1*, both tumor-suppressor, as miR-182-5p target genes. According to miR-182-5p expression, decreased protein levels were observed in tumors compared to peritumor tissues. Analysis of miR-182-5p, Cyld and Foxo1 expression levels, based on datasets from human HCC samples, showed results consistent with those from our mouse models, and also highlighted the ability of miR-182-5p to distinguish between normal and tumor tissues (AUC 0.83). Overall, this study shows, for the first time, miR-182-5p overexpression and Cyld-Foxo1 downregulation in hepatic tissues and tumors from a diet-induced NAFLD/HCC mouse model. These data were confirmed by the analysis of datasets from human HCC samples, highlighting miR-182-5p diagnostic accuracy and demonstrating the need for further studies to assess its potential role as a biomarker or therapeutic target.

## 1. Introduction

Non-alcoholic fatty liver disease (NAFLD) is considered to be the most relevant chronic liver disease worldwide. A recent meta-analysis estimated a prevalence of 32.4%, which significantly increased over a timeframe of about ten years. Incidence has been reported as 46.9 cases per 1000 person/year. Both prevalence and incidence are significantly higher in men than in women, with an overall prevalence of 39.7% vs. 25.6% and an incidence rate of 70.8 vs. 29.6 cases per 1000 person/year in men and women, respectively [1]. A variable percentage of NAFLD patients progress from steatosis, characterized by a high accumulation of triglycerides in hepatocytes, to the more severe steatohepatitis (NASH) with inflammation and a possible increase in liver damage, which leads to fibrosis, the onset of cirrhosis and, eventually, hepatocellular carcinoma (HCC) [2]. It has been described that NAFLD patients who develop NASH and cirrhosis have a higher risk of developing HCC (cumulative incidence between 2.4% over 7 y and 12.8% over 3 y) [3]. General NAFLD-predisposing factors include sedentary lifestyle and excessive caloric intake. In this regard, we demonstrated in previous studies in a NAFLD mouse model that not only a high-fat (HF), but also a long-term hypercaloric low-fat/high-carbohydrate (LF-HC) diet, is able to initiate the disease and promote its progression through the characteristic stages up to HCC development [4,5,6]. Inflammation is one of the main processes triggering NAFLD [7,8,9] in addition to the impairment of DNA damage/repair mechanisms and oxidative stress [10,11,12,13,14]. Genetic factors, particularly genetic polymorphisms, which appear to be involved in NAFLD (e.g., the patatin-like phospholipase domain-containing 3 (*PNPLA3*) I148M variant and transmembrane 6 superfamily member 2 (*TM6SF2*) E167K variant) have also been described [15,16]. At the epigenetic level, microRNAs (miRNAs) are considered important modulators of post-transcriptional gene regulation. They constitute a family of short, non-coding RNAs that are able to interact with the 3′UTR of target mRNAs to negatively regulate their expression, thus playing a pivotal role in fine-tuning fundamental physiological and pathophysiological processes (e.g., cell differentiation, proliferation, programmed death and metabolism). It is known that one mRNA can be targeted by multiple different miRNAs and one miRNA can target multiple different mRNAs [17,18]. In this context, starting from analyses on a C57BL/6J NAFLD mouse model fed with a long-term HF or LF-HC diet, we identified a panel of 15 miRNAs modulated throughout the progression of the disease up to HCC development [4,5]. Among these, miRNA-182-5p emerged as being dysregulated early and maintaining this trend in NAFLD progression. Moreover, bioinformatics analysis evidenced the tumor-suppressors *Cyld* and *Foxo1* as putative miR-182-5p target genes. In this study, we aimed at deepening our understanding of the expression levels and functional relationships between miR-182-5p and Cyld-Foxo1 in hepatic tissues obtained from mouse models affected by HF- or LF-HC-diet-induced NAFL/NASH/HCC progression.

## 2. Results

### 2.1. miR-182-5p Is Overexpressed in NAFLD Mouse Liver Tissues and Tumors

**Figure 1:**
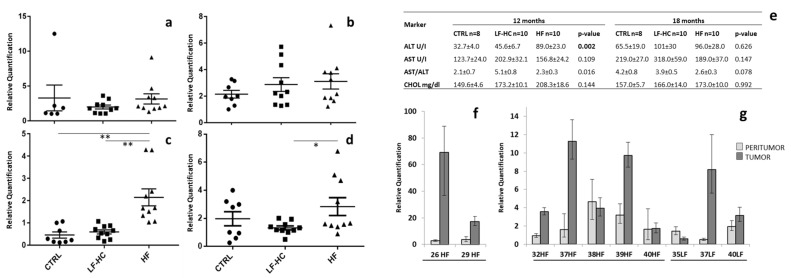
MiR-182-5p expression levels in hepatic non-tumor tissues from standard diet-SD (CTRL, black circle), low-fat/high-carbohydrate (LF-HC, black square) and high-fat (HF, black triangle) fed mice after 3 (**a**), 6, (**b**), 12 (**c**) and 18 (**d**) months. The same reference sample was used for all the comparisons. Plasma levels of alanine aminotransferase (ALT), aspartate aminotransferase (AST) and cholesterol (CHOL) in 12- and 18-month SD (CTRL)-, LF-HC- and HF-diet-fed animals [5] are also reported (**e**). Values are mean ± SEM. Statistical significance was assessed by Kruskal–Wallis test followed by Bonferroni correction (*p*-value < 0.006 in bold). MiR-182-5p expression levels in tumors from 12-month HF (**f**) and 18-month HF and LF-HC mice (**g**), compared to peritumor hepatic tissues. Only statistically significant differences are reported and marked with asterisk(s): ** *p* < 0.005, * *p* < 0.05 (*t*-test).

MiR-182-5p expression levels were analyzed in hepatic tissues from HF-, LF-HC- or standard-diet (SD)-fed mice through the progression of liver disease, and in tumor- compared to non-tumor-adjacent tissues. As shown in Figure 1a–d, a trend towards higher miR-182-5p expression was detected in hepatic tissues from HF-fed and, less marked, from LF-HC mice with respect to those from SD-fed mice, with significant differences after 12 and 18 months. Of note, an unexpected variability in miR-182-5p expression levels was observed in SD-fed mice after 18 months, which is likely attributable to the possible occurrence of deteriorating conditions in terms of liver damage, such as mild steatosis, fibrosis and inflammation due to aging, as previously described [5,19,20]. Consistent with this hypothesis, a previously performed analysis of plasma biomarkers, including alanine aminotransferase (ALT), aspartate aminotransferase (AST) and cholesterol [4,5], showed a significant ALT increase in HF and, to a lesser extent, in LF-HC mice compared to SD-fed animals after 12 months (*p* = 0.002), while there were no differences in ALT, AST and cholesterol levels among the three groups after 18 months (Figure 1e). Interestingly, AST/ALT ratios were ≥2 in all experimental groups, particularly after 18 months.

Overall, an AST/ALT ratio greater than 2 was reported in fasted 54–56-week-old SD/SD + HF-diet-fed and in non-fasted 56–70-day-old SD-fed C57BL6/J mice. Furthermore, in humans, this can be attributed to acute alcoholic hepatitis or advanced fibrosis and cirrhosis in advanced chronic liver disease [5]. The analysis of tumor tissues revealed miR-182-5p overexpression in five out of seven tumors (71.4%) developed in HF mice, and in two out of three (66%) tumors in LF-HC mice (Figure 1f,g). Globally, results suggest that the miR-182-5p expression level increase, detected principally in HF-fed animals, could be correlated to the severity of the disease, since an HF diet was demonstrated to induce faster NAFLD progression than an LF-HC diet, although the latter was able to cause very similar levels of liver damage later on [5].

### 2.2. Cyld and Foxo1 Are miR-182-5p Target Genes

**Figure 2:**
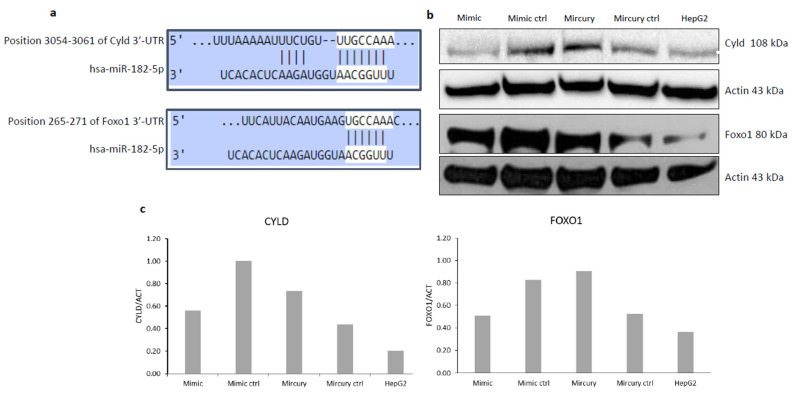
MiR-182-5p sequence alignment to Cyld/Foxo1 3′-UTR (modified from TargetScan v.8) (**a**). Cyld/Foxo1 expression in HepG2 cells after transfection (72 h) with miR-182-5p mimic, antagomir (MiRCURY) and related controls (ctrl) (**b**). Densitometric analysis of immunoblotting is also shown (**c**).

**Figure 3:**
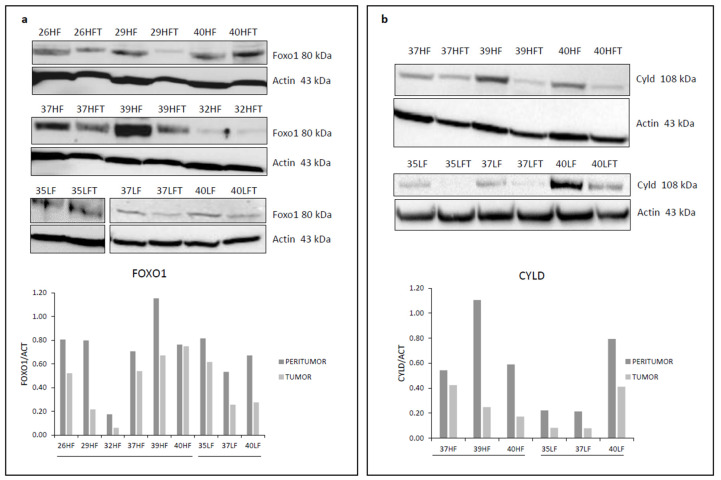
Foxo1 (**a**) and Cyld (**b**) protein expression in liver peritumor (HF, LF) and tumor (HFT, LFT) tissues. Numbers are for identifying individual mice. Densitometric analysis of immunoblotting is also shown. Due to the small amount of tumor tissue, it was not possible to assay some samples (i.e., 26HFT, 29HFT and 32HFT for Cyld; 38HFT for Cyld and Foxo1).

Bioinformatics analysis predicted *Foxo1* (Diana miRpath v3.0) and *Cyld* [21] to be miR-182-5p target genes (Figure 2a). In order to ascertain the interaction between miRNA and target mRNA, Cyld/Foxo1 protein expression after the transfection of HepG2 liver cancer cells with an miR-182-5p mimic or antagomir was tested. The results show protein downregulation in the presence of the miR-182-5p mimic and, conversely, upregulation in the presence of the antagomir, demonstrating miR-182-mediated Cyld/Foxo1 regulation (Figure 2b,c). Analysis of Cyld and Foxo1 mRNA expression levels, performed by RT-PCR, globally revealed no relevant differences in peritumor compared to tumor tissues obtained from HF- and LF-HC-fed mice (Supplementary Figure S1). However, according to the post-transcriptional regulation activity of miRNAs, immunoblot analysis confirmed overall Cyld and Foxo1 protein downregulation in tumor compared to adjacent non-tumor samples (Figure 3).

Immunohistochemistry (IHC) analysis confirmed the immunoblot results in a subset of samples for which sufficient material was available (Supplementary Figure S2).

### 2.3. miR-182-5p Is Upregulated While CYLD and FOXO1 Are Downregulated in Human HCC Samples

**Figure 4:**
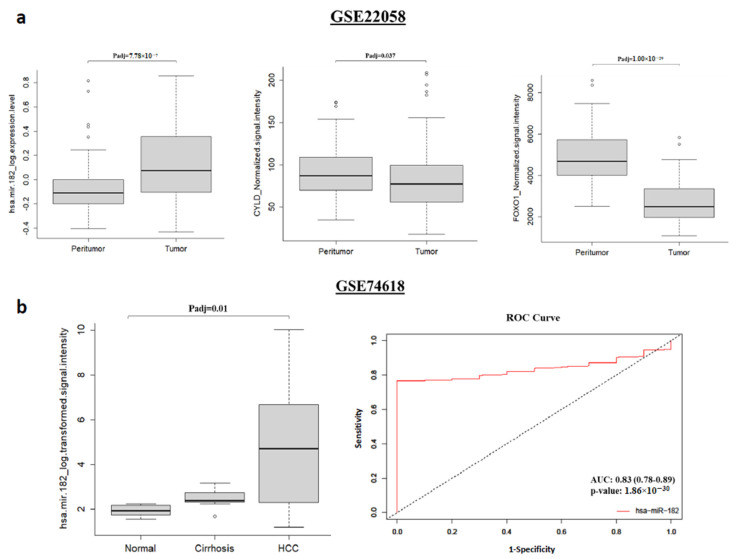
Boxplot showing miR-182-5p, Cyld and Foxo1 expression in peritumor and tumor tissues from 96 HCC patients (GSE22058 datasets) (**a**). Boxplot showing miR-182-5p expression in normal (n = 10), cirrhotic (n = 10) and tumor (n = 218) tissues (GSE74618 dataset); ROC curve analysis of miR-182 (normal vs. HCC) is also reported (**b**). The area under the curve (AUC) with a corresponding 95% confidence interval (CI) and *p*-value was used as the main parameter to assess the diagnostic potential of miR-182-5p.

To deepen the involvement and possible role of miR-182-5p in HCC pathogenesis, we investigated its expression in human normal vs. tumor tissues using publicly available datasets. A statistically significant difference in miR-182-5p expression was found in 8 out of 11 HCC studies selected from the Database of Differentially Expressed miRNAs in Human Cancers (dbDEMC), for a total of 1135 tumors and 550 normal tissues analyzed (Supplementary Table S1). Evaluation of miR-182-5p, Cyld and Foxo1 expression levels in peritumor vs. tumor hepatic tissues (GSE22058 datasets), based on the GEO2R tool, demonstrated not only significant miR-182-5p upregulation, but also concomitant Cyld and Foxo1 downregulation in cancer samples (Figure 4a). By using the GEO2R tool, miR-182-5p expression levels were also assessed in normal, cirrhosis and tumor tissues (GSE74618 dataset). As shown in Figure 4b, significant upregulation of miR-182-5p was detected in HCC samples, and a trend towards a progressive miR-182-5p expression increase from normal to cirrhosis up to cancer development was also observed. Considering the ROC curve, miR-182-5p showed very good diagnostic accuracy, with an AUC of 0.83 (CI: 0.78-0.89; *p*-value of 1.86 × 10^−30^), highlighting the ability to distinguish between normal and tumor hepatic tissue samples with high accuracy, and suggesting that this miRNA could potentially be used as a tumor and/or diagnostic biomarker in HCC. Overall, analyses of miR-182-5p, Cyld and Foxo1 expression levels in publicly available datasets in human HCC showed consistent results with those obtained from our experiments with liver tissues and tumors from NAFLD/HCC mouse models.

## 3. Discussion

We previously demonstrated NAFL-NASH-HCC progression in mouse models under a long-term HF- or LF-HC diet. Both diet regimens were able to induce, with different timings, very similar liver tissue damage that led to tumor development [5,6]. MiRNA expression analysis on such in vivo models also highlighted global and early miR-182-5p overexpression in hepatic tissues from HF- and LF-HC-fed animals, and in HF/LF-HC tumors with respect to peritumor tissues [4]. In this study, we further confirmed miR-182-5p expression in HF/LF-HC mouse hepatic tissues in comparison to SD-fed animals.

Notably, we observed an unexpected variability in miR-182-5p expression levels in SD-fed mice after 18 months, which is likely attributable to possible deteriorating conditions in terms of liver damage, most likely due to aging [19,20].

In fact, as previously described in the literature, it was observed that aging can increase the susceptibility to acute liver injury as well as the fibrotic response. Aging was also associated with the severity and poor prognosis of several hepatic diseases, including NAFLD [20]. According to the inflamm-aging theory, aging appears also to favor NAFLD/NASH/HCC progression, although this relationship has not been fully elucidated [19]. Gregg et al. [22] used Ercc1(−/Δ) mice as a model of accelerated aging driven by a DNA repair defect to study aging-related liver changes. Among the different features of liver damage, they observed areas of necrosis, foci of hepatocellular degeneration and acute inflammation as well as a loss of hepatic architecture, fibrosis and steatosis in both 5-month-old Ercc1(−/Δ) mice and 24-36-month-old wild-type mice, highlighting not only the parallelism between accelerated aging driven by DNA repair defects and normal aging, but also a series of histopathological and functional aging-related liver changes. Therefore, it is plausible that interindividual differences coupled with aging may further favor NAFLD-related conditions. However, it is evident that specific diets play a crucial role in promoting the pathological progression up to HCC onset, as demonstrated by the tumor formation in 12/18-month HF-fed and 18-month LF-HC-fed mice and, conversely, by a lack of tumors in SD-fed mice.

MiR-182, miR-183 and miR-96 belong to a polycistronic miRNA cluster on murine chromosome 6q. The orthologous region in *H. sapiens* is located on chromosome 7q32.2, with the miRNAs arranged in the same order. A meta-analysis of the miRNA-183 family in human cancers, comparing tumor with non-tumor tissues, showed consistent miR-182-5p upregulation in 15 cancer types (among them, breast, colorectal, bladder and prostate cancer, as well as HCC) [23], suggesting a putative role as a prognostic marker or therapeutic target. With respect to HCC, miR-182-5p was found to be upregulated in mouse models of TCE (trichloroethylene)- and DEN (diethylnitrosamine)-induced hepatocarcinogenesis [21,24] as well as in HCC cells under hypoxic conditions, thus promoting angiogenesis [25]. A circulating miR-182-5p increase was shown in HCC patients [26,27], and miR-182 sponging was described to induce tumor suppressor activity in HCC cells [28,29,30]. Several target genes with tumor-suppressor properties have been described for miR-182-5p in HCC, such as *metastasis suppressor 1*, *Cepba, EphrinA5*, *Foxo3A* and *programmed cell death 4* [31,32,33,34,35].

Here, we also focused on the identification of possible miR-182-5p target genes, highlighting miR-182-mediated Cyld and Foxo1 downregulation in our diet-induced models.

These two genes are known as tumor suppressors and have been described in several types of cancer, such as prostate [36,37,38,39], colorectal [40,41,42] and breast cancer [43,44,45], as well as in HCC [46,47,48,49]. *Foxo1* is a member of the widely expressed Foxo transcription factor family, which also includes *Foxo3/4/6*. Foxo proteins are targets of the IGF-1 biochemical pathway and can be regulated by the PI3K/PKB phosphorylation pathway, with subsequent translocation from the nucleus to the cytoplasm, thus blocking transcription and playing a role either as a tumor suppressor or, in several circumstances, as tumorigenic factors [50]. The high expression levels of Foxo1 are detected in liver and pancreas. Regarding HCC, Foxo1 shows tumor suppressor activity by resisting precancerous oxidative stress [51] and inhibiting cell migration and invasion [52]. *Foxo1* is considered a target gene of several miRNAs, such as miR-1269 [53], miR-3174 [47], miR-300 [54] and miR-182 [55]. Concerning the last one, the miR-182/Foxo1 interaction was demonstrated only in in vitro cell systems; in this regard, our work adds considerable evidence, showing contextual miR-182-5p overexpression and Foxo1 reduction in mouse models.

Cyld is a deubiquitinase that mainly acts through the hydrolysis of K63- and M1-linked ubiquitin chains [56]. Cyld negatively regulates NF-kB, known to be one of the most important players in inflammation and liver cancer [57], and the MAPK signaling cascade [58,59] by removing ubiquitin chains from signaling molecules, such as NEMO, TRAF2, TRAF6, TRAF7, TAK1 and RIP1 [60]. Moreover, Cyld induces the decrease in Wnt/β-catenin signaling activity by disheveled deubiquitination [61] and is considered an important regulator of necroptosis [62]. Furthermore, Nikolau et al. [63] demonstrated that Cyld is involved in the regulation of hepatocyte homeostasis and that its inactivation causes inflammation, fibrosis and cancer through chronic activation of the TGF-beta-activated kinase (TAK1) and c-Jun N-terminal kinase (JNK). Cyld downregulation and involvement in HCC development were also demonstrated in human samples [64] and in a mouse model with liver-specific *Cyld* exon 7/8 deletion [65]. Several miRNAs, including miR-362-5p, miR-501-5p and miR-922, have been described to be Cyld-negative regulators in HCC [66,67,68], and miR-182-mediated Cyld regulation has been demonstrated in glioma [69] and in gastrointestinal stromal tumors [70], highlighting the possible role of a specific antagomir as a promising therapeutic strategy [71,72]. Interestingly, a study by Xu et al. [73] reported that the use of anti-miR-182 was able to restore the expression of several cell-cycle genes, including *Cyld* and *Foxo1*, in a mouse model of an orthotopic ovarian cancer xenograft. To date, there is no information about miR-182-mediated Cyld regulation in HCC; here, for the first time, we demonstrated the miRNA–mRNA relationship, providing the basis for the potential use of new anti-miR-182-5p therapeutic approaches in HCC.

In fact, the characterization of miRNAs and the related specific target genes in cancer can improve our understanding of their role in tumorigenesis, not only for deepening biological mechanisms, but also in terms of potential new therapeutic targets [74] and/or biomarker identification [75]. NAFLD is a widespread liver chronic disease, the progression of which has been demonstrated to increase the risk of terminal hepatic conditions, such as cirrhosis and cancer [76]. In addition, liver cancer is one of the most commonly diagnosed cancers lately. We demonstrated that miR-182-5p expression is dysregulated early in a mouse model of diet-induced NAFLD/NASH/HCC progression and increased in tumor compared to peritumor liver tissues. Contextually, decreased protein levels of Cyld and Foxo1 tumor suppressors were detected, and the in vitro results indicated miR-182 mediated Cyld and Foxo1 regulation.

To reinforce this, the investigation of miR-182-5p expression in publicly available datasets from the dbDEMC database showed a significant expression level difference in 8 out of 11 studies based on the comparison between human normal and tumor liver tissues; notably, all these works reported miR-182-5p upregulation in HCC samples, thus highlighting consistent results between studies characterized by series of different sizes and analysis methods. Furthermore, the expression levels of miR-182-5p, Cyld and Foxo1 detected in liver human samples by using GSE22058 datasets evidenced the miR-182-5p expression increase in hepatic disease progression associated with a concurrent Cyld/Foxo1 level decrease.

We also observed a trend towards a progressive increase in miR-182-5p expression in normal, cirrhosis and human HCC samples. Cirrhosis is the main risk factor for the development of HCC; therefore, it can be hypothesized that the miR-182-5p expression increase, starting in the precancerous forms, may be related to the progression to malignant disease. Interestingly, the upregulation of miR-182-5p in patients with NAFLD-related fibrosis as well as in tissues from hepatitis C patients with advanced fibrosis compared to patients with early fibrosis was previously observed through high-throughput sequencing analysis [77,78]. Taken together, these data provide new insights into the underlying molecular mechanisms and possible involvement of miRNAs in the initiation and progression of liver diseases.

Lastly, ROC analysis showed miR-182-5p’s capability to distinguish between normal and cancerous HCC tissues, encouraging additional studies to confirm the possible role of miR-182-5p as a diagnostic biomarker.

The key role of miR-182-5p in liver-related diseases [21,23,24] and, more importantly, the suppressor activity in HCC cells induced by its inhibition make this miRNA an interesting and promising therapeutic target [28,29,30], with a possibility of clinical applicability thanks to the new development of regulatory guidelines and applications in pharmacological drug delivery and preclinical toxicology [79,80].

At the same time, miR-182-5p represents a potential diagnostic biomarker due not only to its ability to discriminate between tumor and normal samples with high accuracy, but also due to its ability to discriminate between precancerous and cancerous conditions. Overall, the stable expression and broad spectrum of functions of miRNAs in human cancers make them promising candidate biomarkers for early diagnosis [81].

Undoubtedly, our results do not support the immediate use of miR-182-5p in the clinical setting: further investigations in large case–control series and rigorous trials in selected patient cohorts are therefore essential to evaluate the clinical applicability of miR-182-5p.

In conclusion, this study shows, for the first time, miR-182-5p overexpression and related Cyld and Foxo1 downregulation in hepatic tissues and tumors obtained from diet-induced NAFLD/HCC mouse models, paving the way for further studies to assess the potential role of miR-182-5p as a biomarker or therapeutic target. Since miRNAs are considered suitable circulating biomarkers as well, it would be useful to analyze miR-182-5p levels in sera from patients at different NAFLD stages of progression.

## 4. Materials and Methods

### 4.1. Mouse Models

In this work, samples that were already available, as they were collected during previous studies on mouse models [4,5], were used. To summarize briefly, C57BL/6J mice were purchased from Charles Rivers Laboratories (France) and maintained at 21 °C on a 12 h light–dark cycle. Twenty-day-old male mice were randomly split into 8 groups (10 animals each), including 4 groups fed with a high-fat (HF) diet and 4 groups fed with a low-fat and high-carbohydrate (LF-HC) diet for 3, 6, 12 and 18 months, as previously described. Control mice groups (8 animals each) that were fed with a standard diet (SD) for 3, 6, 12 and 18 months were also included in the study. Mice were sacrificed by CO_2_ asphyxiation, and, through laparotomy, the liver was visualized and rapidly excised. Liver tumors were also excised, counted and measured. Overall, tumors were detected in 20% (2/10) and 50% (5/10) of HF-fed mice after 12 and 18 months, respectively, and in 30% (3/10) of LF-HC-fed animals after 18 months [5].

### 4.2. RNA Extraction and miR-182 Expression Analysis

RNA was extracted from mouse liver tissues and tumors, recovered after dissection, and stored in RNAlater stabilization solution (Ambion, Thermo Fisher Scientific, Waltham, MA, USA) at −80 °C by using the miRVana microRNA isolation kit (Life Technologies, Thermo Fisher Scientific, Waltham, MA, USA) according to the manufacturer’s instructions. The expression profile of miR-182-5p was investigated by quantitative Real-Time PCR (qRT-PCR) using the TaqMan miRNA Assay (Life Technologies). Starting from 10 ng of total RNA input, reverse transcription was performed using the TaqMan MicroRNA Reverse Transcription kit (Life Technologies) by following the manufacturer’s protocol, and qRT-PCR, consisting of 10 min at 95 °C and 40 cycles of 15 s at 95 °C and 60 s at 60 °C (TaqMan Universal Master Mix II, no UNG protocol, Life Technologies), was carried out on a ViiA7 platform (Applied Biosystem, Thermo Fisher Scientific, Waltham, MA, USA).

Commercially available TaqMan assays for the target (miR-182, ID 002599) and endogenous control (U6snRNA, ID 001973) were used (Life Technologies). The same reference control sample (ctrl33) was used for all comparisons as a calibrator. Each sample was run in triplicate and the 2^−ΔΔCt^ method was applied to determine the relative miR-182-5p expression levels, based on the following steps: ΔCt = Ct _target miRNA_ − Ct _endogenous control_, ΔΔCt = ΔCt _sample_ − ΔCt _reference sample_, and relative quantification (RQ) = 2^−ΔΔCt^. Error bars were constructed using RQmin and RQmax values (range of possible RQ values defined by the standard error of ΔCt: lower/upper limit of the confidence interval). Data analysis, described above, was carried out automatically using QuantStudio Real-Time PCR software v1.3 (Applied Biosystems). Statistical analyses were performed using GraphPad Prism 6.

### 4.3. Transfection of miRNA Mimic and miRNA Inhibitor

Human hepatocellular carcinoma cell line HepG2 (ATCC) was cultured in RPMI medium (Euroclone, Milan, Italy), supplemented with 10% FBS, 1% PES and 1% glutamine, and maintained in a humidified atmosphere at 37 °C with 5% CO_2_.

Cells were then cultured in 60 mm dishes until reaching a 30–50% confluence, and then were transfected by using the siRNA INTERFERIN transfection reagent (Polyplus, Illkirch-Graffenstaden, France) according to the manufacturer’s instructions. MiR-182-5p overexpression was obtained by using the CONmiR mimic (RIBOXX) HSA-miR-182-5P (5 nmol) and, as a control, the CONmiR mimic NEGATIVE-CONTROL N1 (2 nmol). For inhibition, the miRCURY LNA miRNA INHIBITOR HSA-miR-182-5P (5 nmol) and miRCURY LNA miRNA INHIBITOR CONTROL (5 nmol) were used as the antagomir and control, respectively. The mimic and miRNA inhibitor were used at a final concentration of 50 nM. Cells were harvested after 24, 48 and 72 h, and then were centrifuged and stored at −80 °C before carrying out the protein extraction.

### 4.4. Western Blot Analysis

Immunoblotting was performed to evaluate the expression of Cyld and Foxo1 in HepG2 cells, as well as in frozen liver peritumor (HF, LF-HC) and tumor (HFT, LFT) tissues, after transfection with the miR-182-5p mimic and antagomir. HepG2 cells were lysed in 60 µL modified RIPA Buffer (PBS 1X, NP40 1%, sodium deoxycholate 0.5%, SDS 1%, Complete-Mini protease inhibitor cocktail tablet (Roche Diagnostics, Basel, Switzerland), PMSF 10 mg/mL, aprotinin 10 mg/mL and sodium orthovanadate 0.1 M (Sigma-Aldrich, Burlington, MA, USA)). Tissue samples were homogenized in 500 µL of RIPA Buffer by TissueLyser LT and 5 mm beads (Qiagen, Hilden, Germany) at 50 Hz for 3 min.

Cell lysates were incubated on ice for 30 min and then centrifuged at 14,000× *g* for 30 min at 4 °C. The supernatant was recovered and stored at −80 °C until use. Protein concentration was determined using the standard BCA control with the Pierce BCA protein assay kit, according to the manufacturer’s instructions.

A total of 30 µg of protein extracts was loaded onto a SDS-PAGE and subjected to electrophoresis; then, proteins were transferred to a nitrocellulose membrane by using IBlot 2 NC Regular Stacks (Invitrogen, Thermo Fisher Scientific, Waltham, MA, USA) and were hybridized overnight at 4 °C with the rabbit anti-Cyld (#8462, Cell Signaling Technology, Danvers, MA, USA) and the rabbit anti-FoxO1 (#c29H4, Cell Signaling Technology; # 4370, Cell Signaling Technology, Danvers, MA, USA) monoclonal antibodies.

The membrane was washed in TBS-T and incubated for one hour at room temperature with the goat anti-rabbit IgG-HRP secondary antibody (sc-2030, Santa Cruz Biotechnology, Dallas, TX, USA). Actin (sc-1615, Santa Cruz Biotechnology, Dallas, TX, USA) was used as the endogenous control. The chemiluminescent detection system (SuperSignal™ West Pico Plus, Thermo Scientific, Waltham, MA, USA), ChemiDoc XR + (Bio-Rad, Hercules, CA, USA) system or autoradiography films were used to detect signals. Bands corresponding to proteins of interest were scanned and quantified by densitometry using ImageJ software (https://imagej.nih.gov/ij/, accessed on 17 April 2023).

### 4.5. Publicly Available Datasets

The Database of Differentially Expressed miRNAs in Human Cancers (dbDEMC, https://www.biosino.org/dbDEMC/index, accessed on 10 January 2023) was queried to evaluate miR-182-5p expression in HCC patients. Studies were selected based on the following criteria: (i) all available in public repositories, including in the Gene Expression Omnibus (GEO), the Sequence Read Archive (SRA), ArrayExpress and The Cancer Genome Atlas (TCGA); (ii) data from high-throughput analysis (microarray/miRNA-sequencing); and (iii) differential expression analysis in normal compared to tumor samples. Overall, 11 HCC studies were selected (GSE10694, GSE147889, GSE21362, GSE22058, GSE36915, GSE39678, GSE6857, GSE74618, E_MTAB_4170, SRP049590/GSE63046 and TCGA_LIHC datasets) for a total of 1310 tumor samples, 735 normal samples and 10 cirrhotic tissue samples.

Among these, GSE22058 datasets were used to investigate the expression levels of miR-182-5p, Cyld and Foxo1 in peritumor and tumor tissues from 96 HCC patients (platform GPL10457 for miRNA analysis and platform GPL6793 for mRNA analysis), while the GSE74618 dataset was used for expression analysis of miR-182-5p in normal (n = 10), cirrhotic (n = 10) and tumor (n = 218) tissues. Gene expression data were analyzed using GEO2R software (R 3.2.3, Biobase 2.30.0, GEOquery 2.40.0, limma 3.26.8); an adjusted *p*-value (padj) ≤ 0.05 (Benjamini–Hochberg FDR correction for multiple testing) was considered statistically significant.

The receiver operating characteristic (ROC) curve was carried out to determine the miR-182-5p diagnostic value by calculating the area under the curve (AUC) with a 95% confidence interval (CI) using the easyROC web-tool [82]; the predictive ability of the model was based on AUC values that were considered to be excellent (0.9–1.0), very good (0.8–0.9) or good (0.7–0.8) [83].

### 4.6. Clinical Chemistry Assays

The clinical chemistry assays and related results were already described in [5].

## Data Availability

The original contributions presented in the study are included in the article. Further inquiries can be directed to the corresponding author. Publicly available datasets analyzed in this study are available in the dbDEMC database (https://www.biosino.org/dbDEMC/index, accessed on 10 January 2023) and GEO repository (https://www.ncbi.nlm.nih.gov/geo/query/acc.cgi?acc=GSE22058, accessed on 12 January 2023), (https://www.ncbi.nlm.nih.gov/geo/query/acc.cgi?acc=GSE74618, accessed on 12 January 2023).

## Supplementary Materials

The following supporting information can be downloaded at: https://www.mdpi.com/article/10.3390/ijms24119239/s1.
