# Supplementary material for: MiR-182-5p Is Upregulated in Hepatic Tissues from a Diet-Induced NAFLD/NASH/HCC C57BL/6J Mouse Model and Modulates Cyld and Foxo1 Expression
Source: Int J Mol Sci. 2023 May 25;24(11):9239. doi: 10.3390/ijms24119239 (PMC10252350; doi:10.3390/ijms24119239)

Supplementary Figure S2. Representative images of Cyld and FoxO1 immunohistochemistry (primary antibodies: cylindromatosis 1 (E-10), sc-74435 and FKHR (C9), sc-374427 - Santa Cruz Biotechnology) in HF and LF-HC liver normal and tumor tissues. The ABC staining system, Avidin/ Biotin Complex (VECTASTAIN Elite ABC System PK-6100) with DAB substrate (3-3'diaminobenzidine; Ultravision detection System DAB Substrate System, Lab Vision Corporation, TA-125 -HD) were used. Nuclei were counterstained with hematoxylin.

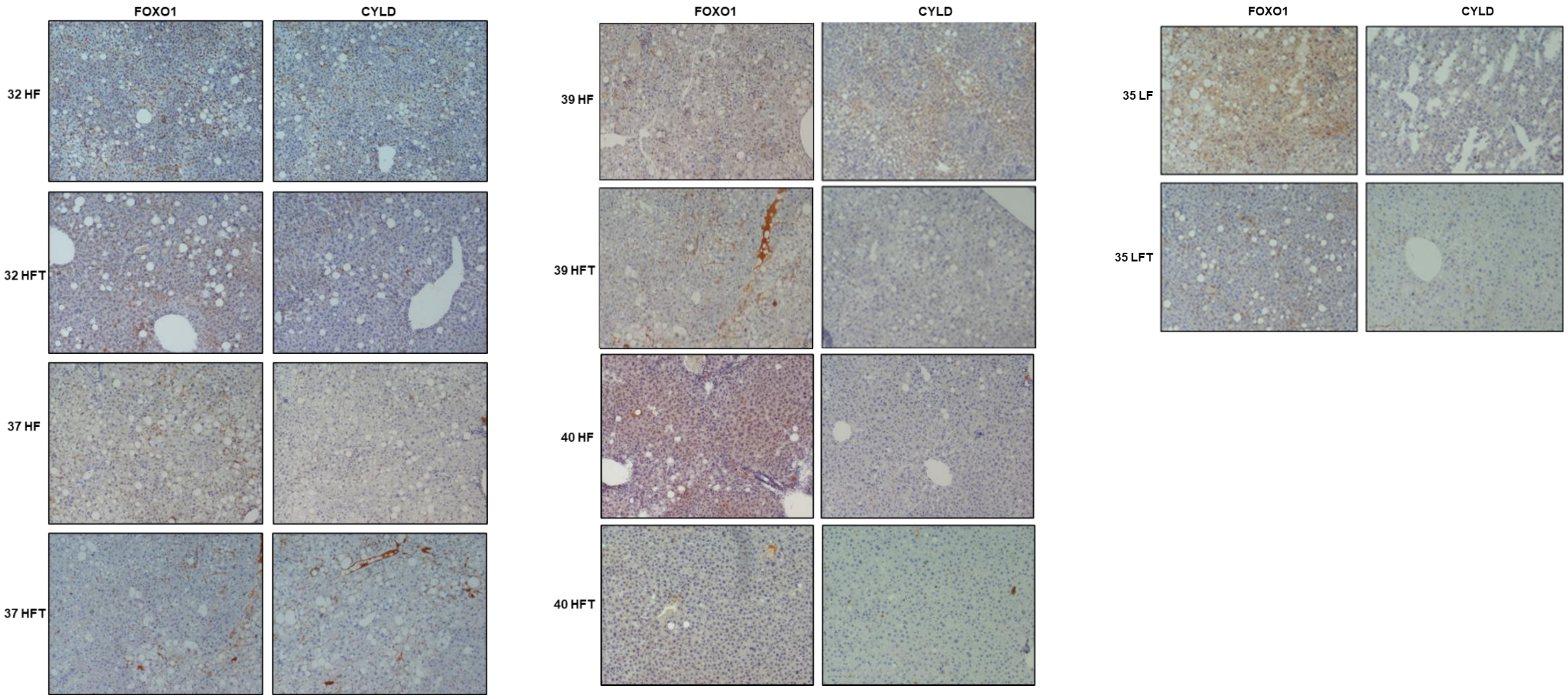

Supplement: Supplementary file 1 [file ijms-24-09239-s001.zip › Figure S2.pdf]
