# Supplementary material for: MiR-182-5p Is Upregulated in Hepatic Tissues from a Diet-Induced NAFLD/NASH/HCC C57BL/6J Mouse Model and Modulates Cyld and Foxo1 Expression
Source: Int J Mol Sci. 2023 May 25;24(11):9239. doi: 10.3390/ijms24119239 (PMC10252350; doi:10.3390/ijms24119239)

Supplementary Figure S1. Analysis of Cyld and Foxo1 mRNA expression levels. Peritumor normal and tumor HF/LF-HC hepatic tissues were retrotranscribed by using the High-Capacity cDNA reverse transcription kit (Thermo Fisher) according to the manufacturer's instructions. One microliter of product was used for semi-quantitative multiplex RT-PCR (Promega) following the manufacturer's protocol (40 sec at 94°C, 30 sec at 60°, 40 sec at 72°C, 35 cycles). Primers' sequences as follows: FOXO1 - sense CCCAGTGTGAATCATGGGCA, α-sense AGACTCAGTTTGTCCAAGCAGAAC; CYLD - sense GCTAATGATTGCCTTGAGAAAGACA, α-sense TGTCTCATACTTAGGAGCCCCT; HPRT - sense TTGGATACAGGCCAGACTTTG and α-sense TGGCAACATCAACAGGACTC. HPRT (hypoxanthine-guanine phosphoribosyl transferase) was used as endogenous control.

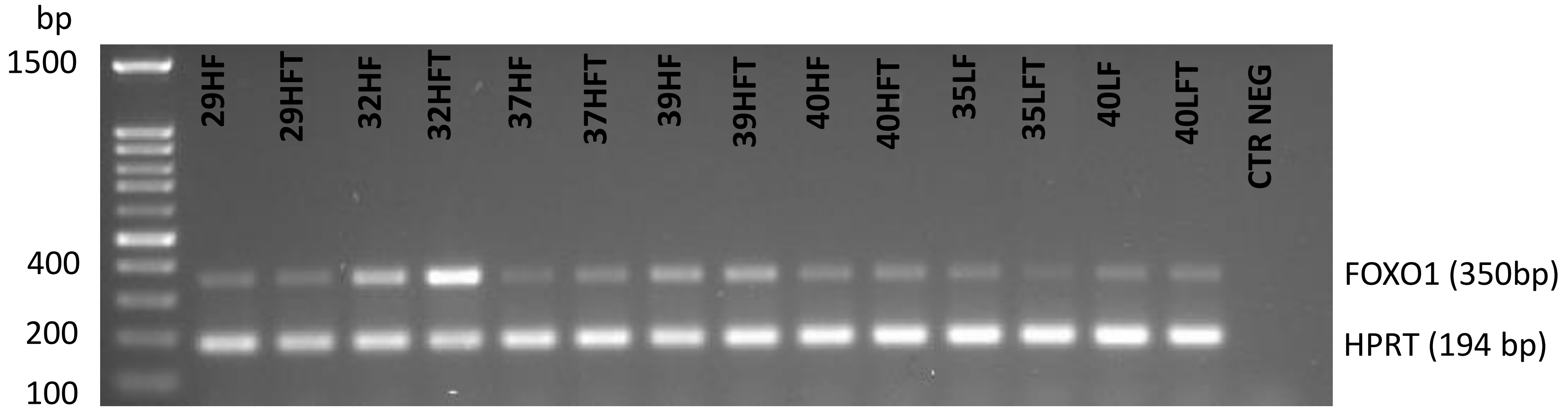

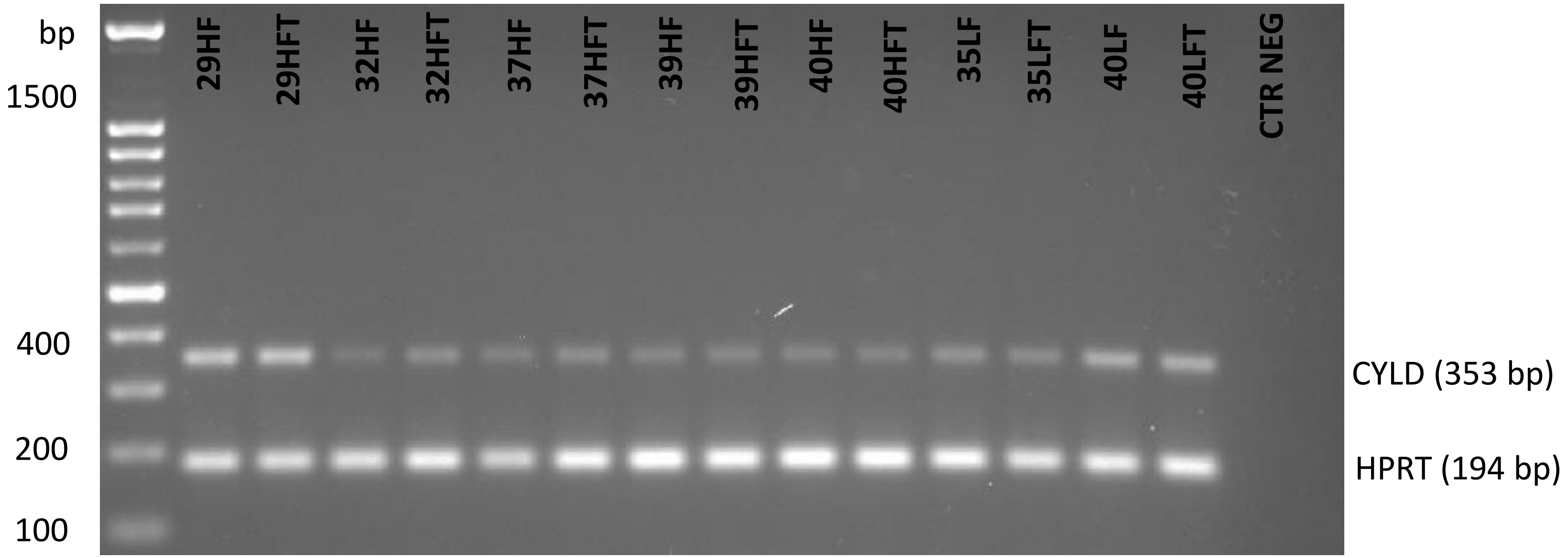

Supplement: Supplementary file 1 [file ijms-24-09239-s001.zip › Figure_S1.pdf]
